# A simple method for tuning the glass transition process in inorganic phosphate glasses

**DOI:** 10.1038/srep08369

**Published:** 2015-02-10

**Authors:** René Fulchiron, Imane Belyamani, Joshua U. Otaigbe, Véronique Bounor-Legaré

**Affiliations:** 1 Université de Lyon, F-69361 Lyon, France; 2 CNRS, UMR 5223, Ingénierie des Matériaux Polymères, F-69621 Villeurbanne, France; 3 Université Claude Bernard Lyon 1, F-69622 Villeurbanne, France; 4 School of Polymers and High performance Materials, The University of Southern Mississippi, Hattiesburg, Mississippi 39406, USA

## Abstract

The physical modification of glass transition temperature (*T_g_*) and properties of materials via blending is a common practice in industry and academia and has a large economic advantage. In this context, simple production of hitherto unattainable new inorganic glass blends from already existing glass compositions via blending raises much hope with the potential to provide new glasses with new and improved properties, that cannot be achieved with classical glass synthesis, for a plethora of applications such as computers screens, glass-to-metal seals, and storage materials for nuclear wastes. Here, we demonstrate that blends of the specific glass compositions studied are miscible in all proportions, an unreported phenomenon in hard condensed matter like glass. Interestingly, excellent agreement was found between the obtained data and calculated *T_g_*s from theoretical equations (Supplementary information) for predicting the composition dependence of *T_g_* for miscible blends with weak but significant specific interactions between the blend components. That this blending method is at present not applied to inorganic glasses reflects the fact that water and chemically resistant phosphate glasses with relatively low *T_g_*s have become available only recently.

In general, when a liquid phase is cooled to below its freezing temperature, it normally transforms into a crystalline solid as Fig. 1 shows^1^,^2^,^3^,^4^,^5^,^6^,^7^. However, inorganic glass liquids like phosphate glasses do not crystallize but instead form a rigid disordered network structure when cooled to low temperatures due to their complex molecular configuration or relatively slow transport. The disordered structure just mentioned is remarkably similar to that of the liquid phase. In contrast, for polymeric liquids, crystallization is complicated because of their monomer chain lengths and weak Van der Waals forces that permit individual chain molecules to readily slide past one another^8^,^9^. It is the complex structural rearrangements required for crystallization that leads to glass formation in both glasses and polymers. It is now generally recognized that inorganic phosphate glasses are polymeric in nature in the sense that they are composed of chain-like or crosslinked structures like organic polymers, albeit the chains (composed of phosphate anion tetrahedra) are much shorter in the former case^10^,^11^,^12^,^13^,^14^,^15^,^16^,^17^.

It is worthy to note that the glass transition temperature (or *T_g_*), which corresponds to the temperature at which a supercooled liquid becomes a glass, is for inorganic glasses what the melting point (or *T_m_*) is for crystalline solids (see Fig. 1). While *T_m_* values do not depend on the direction of the change (i.e., freezing a liquid, melting a solid) or on the change rate, the location of the glass transition region depends on both factors as depicted in Fig. 1. Kovacs showed in 1963 in his classical paper how the *T*_g_ location varies with the cooling rate of ‘glassy' polyvinyl acetate^18^.

It is well known that *T_g_* values are useful for a variety of purposes, especially needed are *T_g_* values as a function of composition for binary polymer blends because they tell us whether the blend components are miscible, or compatible, or not miscible at all. In this context, a single glass transition temperature for all the blends characterizes complete miscibility. On the other hand, compatible systems show two *T_g_* values^19^,^20^ which depend on composition while useful immiscible polymers show that the *T_g_* values for the pure components do not change with composition. Note that the miscibility (or lack of it) is often decisive for all properties for both inorganic glasses and polymers including, for polymers, effects of fillers, nano-confinement effects on segmental motions in polymer composites, and changes of *T_g_* with residual stress^21^,^22^.

While *T_g_* as a function of composition has been used widely to develop useful polymer blends and copolymers, and other simple glassy liquids and thin films from aqueous emulsions and paints, surprisingly it has not been used to develop inorganic glasses with hitherto unattainable *T_g_* values and properties from existing compositions considering the facile and relative technological importance of this approach. The blends behavior and the variation of *T_g_* of silicate glasses with composition have not been systematically studied, probably because of the difficulties of blending them at elevated temperatures (often >1000°C) in the liquid state and of accurately measuring *T_g_* in the very high temperature ranges typical of conventional inorganic glasses. The unique desirable possibility of tailoring *T_g_* of inorganic phosphate glasses via blending should extend the versatile, low cost, and facile blending method to a wide variety of inorganic glass compositions that may lead to new applications such as optoelectronic and biocompatible biomedical devices, and storage materials for nuclear wastes^23^ where already existing glasses cannot be used, essentially similar to current practice in the polymer industry.

In this paper we report the first ever successful attempt of blending ‘premade' (or existing) phosphate glass compositions in the liquid phase to obtain new, hitherto unattainable miscible compositions with a different single *T_g_* that varies with the blend composition. The obtained results are analyzed and interpreted within the context of a number of extant theoretical equations that pervade the literature for predicting the glass transition temperatures of binary blends and copolymers (see Supplementary information). Like for organic polymers, the properties of the phosphate glass blends can be expected to be some combination of that of the blend components to a greater or lesser extent depending on the exact compositions. This is a matter for future investigation.

In the current study described in this paper, representative existing low T_g_ tin-phosphorous oxyfluoride and mixed alkali glass systems were used as good model systems because of their ease of preparation and handling, durability to water and chemicals, low working temperatures (low-*T*_g_s compared to other commonly used inorganic glasses), and sensitivity of intermediate-range order to melt-processing conditions^10^,^24^. It is hoped that the interesting results of this study will provide a basis for further exploration of the facile idea of developing new phosphate glass compositions from already existing glass compositions.

Figure 2a–b shows the DSC traces of the second heating for the pure starting phosphate glass (P-glass) compositions and of the first heating for the blend sample LTw0.56, respectively. Two *T_g_* values are clearly noticeable, each of them being due to the solid-state powder of initial components in the blend. After this first heating and the subsequent cooling, the second heating of this blend (Fig. 2c) still shows two *T_g_* values, revealing that the blend is not totally achieved. However, the first *T_g_* is much more discernible than the second one. Additionally, each *T_g_* moved to a different location compared to that shown in Fig. 2a because the P-glass blends are partially mixed and there are two phases with one phase richer in ILT2 (*T_g_* = 178°C) and the other phase richer in IHT2 (*T_g_* = 276°C). For the third heating, practically only one *T_g_* (186°C) was observed (see Fig. 2d). Therefore, it can be concluded that the total intimate mixing of the P-glass blend is nearly achieved. However, it must be recognized that Fig. 2d still shows a small thermal event around 275°C, probably due to a very small part of the remaining phase that is richer in IHT2. Nevertheless, after another holding at 370°C for 2 hours (not shown here) the heating curve does not exhibit this small event anymore and there is only one *T_g_* at practically the same value as shown here (~186°C).

Now considering the other blends in Table 1, Fig. 2e shows the DSC trace obtained for the P-glass blend sample LTw0.24, after holding at 370°C for 2 hours. Clearly this figure shows that only one *T_g_* (245°C) is discernable, indicating again that a completely miscible P-glass blend was achieved. Additionally, Fig. 2f shows the DSC curve of the third heating of the P-glass blend sample LTw0.78 illustrating total miscibility of the blend components as depicted by the unique single glass transition temperature indicated.

Finally, all the *T_g_* values obtained (taken at midpoints of the glass transition region) are plotted as a function of the IHT2 weight fraction in Fig. 3. From this Figure, it can be concluded that the classical Fox Equation (Supplementary equation (1)) fails in describing the *T_g_* variation of the blends of the phosphate glass compositions studied. From the theoretical point of view, this finding leads to the conclusion that specific interactions between the P-glass blend components (ILT2 and IHT2), favorable to their miscibility, are present in the final P-glass blends. Admittedly, the experimental variation shows a slight negative deviation from the simple mixing rule but much lesser than that predicted by the Fox equation which is strictly valid for blends without any specific interactions (Supplementary information). Interestingly, the experimental data trend is well described by the Gordon-Taylor equation (Supplementary equation (2)). The adjustable parameter *K*, obtained by fitting the experimental results to Supplementary equation (2) was found to be 0.883. By analogy with the expression of Lu and Weiss, (Supplementary equations (17), (18), and (14)), this obtained value of *K* which is quite close to 1 leads to the conclusion that some relatively weak but significant specific interactions are present between both components of the P-glass blends studied. The specific interactions just mentioned are ascribed to the well known ionic interactions and liquid state re-ordering of phosphate anion tetrahedral chains present in phosphate glasses as previously reported in the literature by Otaigbe and Beall^10^ and by other glass science researchers^25^. It is noteworthy that strong interactions (if present) would instead lead to a positive deviation from the mixing rule.

In conclusion, this study unambiguously demonstrates that phosphate glass compositions with significantly different *T_g_* values (and therefore properties) can be easily blended under controlled heating conditions in the liquid phase to obtain new phosphate glass compositions with a different single *T_g_* and properties. Interestingly, the obtained results show that the specific phosphate glass compositions used in this study are miscible in all proportions as predicted by a number of empirical and quantitative equations in the literature for interpreting the composition dependence of *T_g_* for miscible blends with relatively weak but significant specific interactions already described (see Supplementary information). It is noteworthy that the observed phenomenon just mentioned is hitherto unreported for hard condensed matter like inorganic glasses. While the facile blending strategy reported here is used widely for organic polymer blends and copolymers, to our knowledge, this is the first published experimental study of application of this strategy to inorganic glasses to obtain new industrially useful inorganic glasses with enhanced benefits from already existing glass compositions, providing a distinctive transformative and complementary alternative method to the traditional relatively cumbersome chemical synthesis of new glasses with new *T_g_* and properties. With additional engagement of the scientific and industrial communities, it is hoped that the current research finding can be accelerated into glass products with enhanced benefits that includes conservation of naturally occurring oxide compounds used in conventional glass synthesis. Conceptually, it may even be possible to use this simple practical approach to tune important glass application properties like chemical durability of phosphate glasses in general, in a manner that is consistent with Einstein's quote, “Everything should be made as simple as possible, but not simpler”^26^. Thus, making the strategy potentially widely applicable. The current paper will guide and facilitate future progress in this important and emerging area.

## Methods

### Materials

A comprehensive literature review revealed a number of patents illustrating the preparation of inorganic glasses with different *T_g_* values based on inorganic oxides and phosphates. The *T_g_* values are related to the type and the stoichiometric molar concentrations of the reactants used. In this study, phosphate glass (P-glass) compositions with two remarkably different glass transition temperatures and compositions were synthesized in the laboratory according to procedures reported elsewhere by Otaigbe and Beall^10^, Sanford and Tick^27^, Beall and Quinn^28^, and Beall and Pierson^29^. Typical compositions of tin fluorophosphate glass (hereinafter denoted as ILT2) and of a mixed alkali phosphate glass (hereinafter denoted as IHT2) compositions were prepared by conventional glass melting and quenching method. In the current study, the ILT2 sample with a molar composition of 10% SnO + 45% SnF_2_ + 42.8% P_2_O_5_ (all expressed as mol%, and optionally containing 2.2% tin pyrophosphate additive) was prepared by heating, in a furnace maintained at 420°C, a mixture of the stoichiometric amounts of the initial raw materials in an appropriate high-temperature crucible for at least 25 minutes. The glass melt was then poured into a steel mold to form a small circular disk that was subsequently annealed at a temperature of 90°C for 2 hours. The obtained glass was then ground into powder to give the ILT2 sample with a T_g_ of approximately 110°C. A similar procedure was used to prepare the IHT2 sample of this study with a molar composition of 50% M_2_O + 2% Al_2_O_3_ + 48% P_2_O_5_ (where M is an alkali such as Na or Li, and all expressed in mol%). The stoichiometric amounts of the initial raw materials of the IHT2 sample were heated at 1000°C for at least 2 hours, and the resulting glass melt was poured into a steel mold to form a circular disk that was subsequently annealed at 260°C for 2 h to give a relatively high Tg of ~294°C. It is worthy to note that these glasses and other similar glasses can be easily prepared and handled as previously reported^10^,^24^.

### Samples preparation

The P-glass samples, in powder form, were simply mixed by weighing the required amounts of each material in a vial and stirring vigorously by hand. The different weight fractions of the used samples are given in Table 1:

### Thermal Analysis

The thermal properties of the samples were acquired using a Differential Scanning Calorimetry Diamond DSC from Perkin Elmer using between 20 and 30 mg of P-glass blend sample in a nitrogen atmosphere following procedures reported in the literature^30^. For the initial starting phosphate glasses (i.e., samples ILT2 and IHT2), two cycles of heating and cooling were applied from ambient temperature to the maximum temperature (*T_max_*) at heating and cooling rates of 30°C/min. These rates are higher than that frequently applied (10°C/min) in standard DSC measurements in the literature because a higher rate is generally suitable for *T_g_* analysis due to the experimental fact that the heat flux signal is enhanced so that the variation of the heat capacity characteristic of the *T_g_* transition is more easily detected. However, these rates must not be too high otherwise the accuracy of the temperature scale can be reduced. Between the heating and the cooling, the sample was held at *T_max_* for 2 minutes. The maximum temperature used was: 200°C for ILT2 and 370°C for IHT2. In fact, for ILT2, when the temperature was increased to more than 200°C, some instabilities of the signal appeared, due to boiling of the sample in the DSC sample pan. For the blend samples, the imposed temperature profiles were slightly different because the intimate mixing of the materials in the DSC sample pan has to be ensured which means that the temperature *T_max_* must be higher than the *T_g_* of both materials, but *T_max_* and the holding time must not be too high to avoid possible desegregation of the ILT2 material from the blend (especially for the highest content of ILT2). Note that the *T_max_*, the holding time at *T_max_*, and the number of heating and cooling cycles applied to the samples in Table 1 were found to give accurate and reproducible results.

### Extant theoretical considerations

Details of the theoretical considerations are given in the Supplementary Information.

## Author Contributions

R.F. and J.U.O. conceived the research and wrote the paper, I.B. prepared the glass samples, R.F. and V.B.-L. performed the measurements, and all authors analysed and discussed the results.

## Supplementary Material

Supplementary Information A simple method for tuning the glass transition process in inorganic phosphate glasses

## Figures and Tables

**Figure 1:**
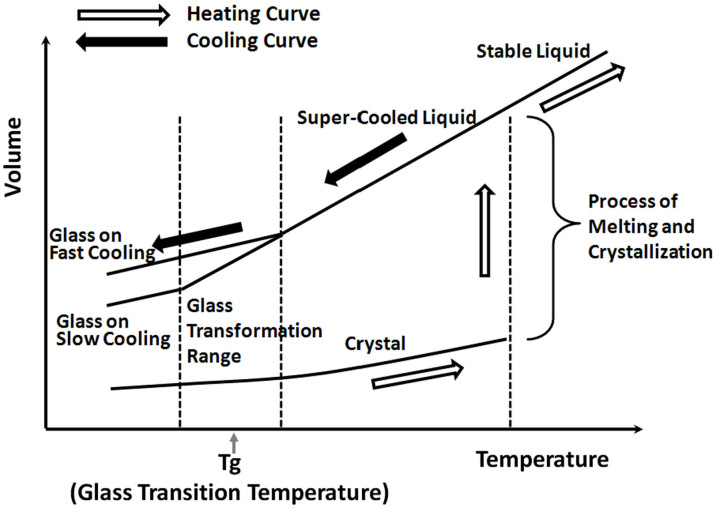
General volume changes associated with heating and cooling in systems susceptible to glass formation (Adapted from Refs. 1–7,1–7,1–7,1–7,1–7,1–7,1–7).

**Figure 2:**
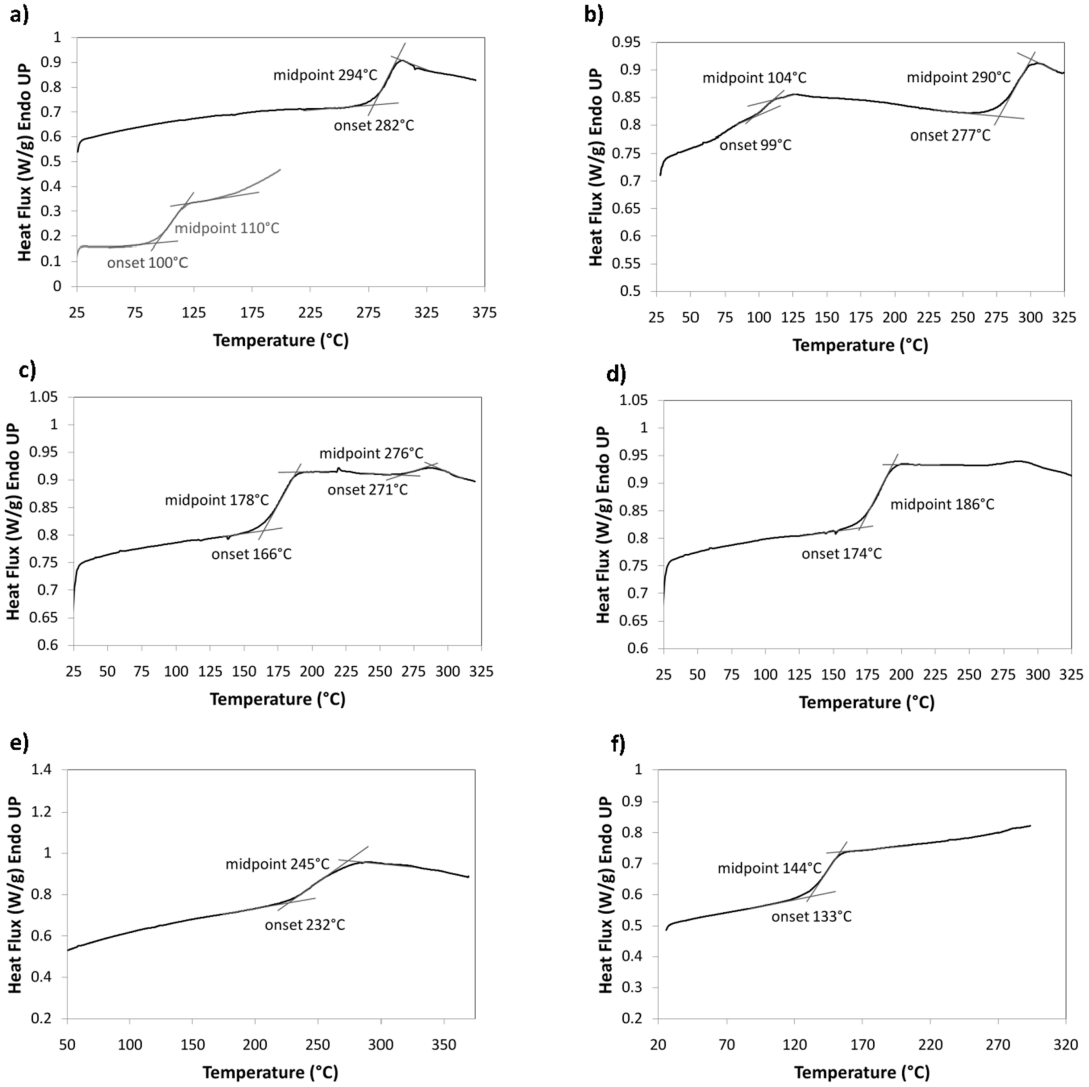
DSC traces: (a) Second heating for starting compositions ILT2 (bottom) and IHT2 (top), (b) first heating for LTw0.56, (c) second heating for LTw0.56, (d) third heating for LTw0.56, (e) second heating after holding at 370°C for 2 hours for LTw0.24, (f) third heating for LTw0.78.

**Figure 3:**
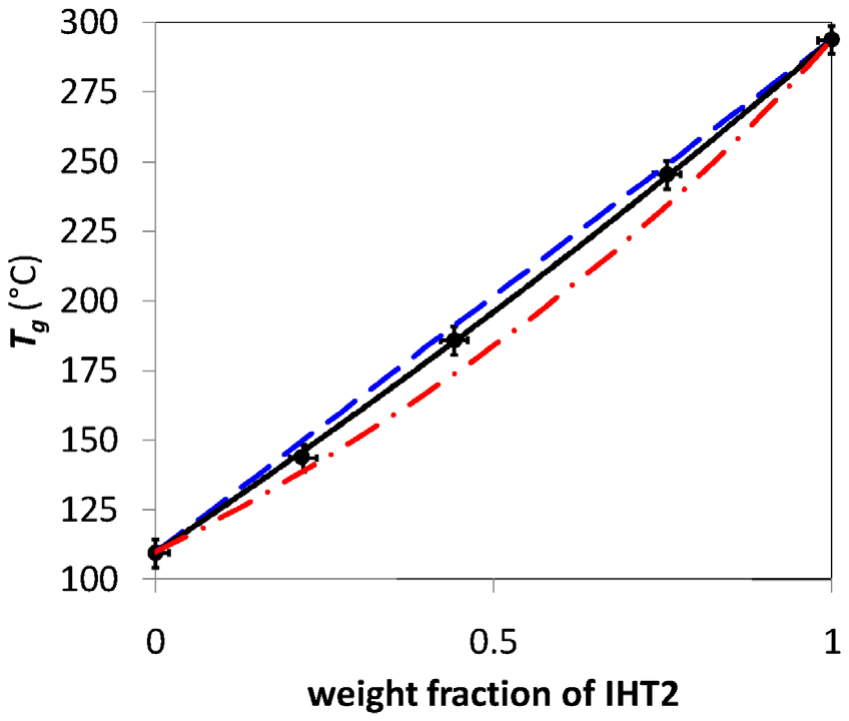
*Tg* versus weight fraction of IHT2. •: Experimental values, – – – – -: Linear mixing rule, —·—·: Fox equation (Supplementary equation (1)),—— Gordon Taylor equation (Supplementary equation (2) with *K* = 0.883). The error bars shown are ±0.01 and ±2°C for weight fraction and *T_g_*, respectively.

**Table 1:** Samples composition and temperature profiles used

Sample name	ILT2	LTw0.78	LTw0.56	LTw0.24	IHT2
Weight fraction of ILT2	1.000	0.783	0.558	0.244	0.000
Weight fraction of IHT2	0.000	0.217	0.442	0.756	1.000
*T_max_* (°C)	200	320	370	370	370
Holding time	2 min	2 min	2 min	2 h	2 min
Number of cycles	2	3	3	2	2

All heating and cooling rates are 30°C/min.
