# Supplementary material for: A simple method for tuning the glass transition process in inorganic phosphate glasses
Source: Sci Rep. 2015 Feb 10;5:8369. doi: 10.1038/srep08369 (PMC4322350; doi:10.1038/srep08369)
Supplement: Supplementary Information — A simple method for tuning the glass transition process in inorganic phosphate glasses [file srep08369-s1.pdf]

A simple method for tuning the glass transition process in inorganic phosphate glasses

René Fulchiron[1,2,3], Imane Belyamani[4], Joshua U. Otaigbe[1,2,3,4*], Véronique Bounor-Legaré[1,2,3]

Extant Theoretical Considerations

Over the past 62 years, a number of theoretical equations have been proposed to predict the composition dependence of T_g for miscible polymer blends and copolymers. As a useful background to interpretation of our obtained results in the main article, the most frequently used equations in practice and cited in the literature are now described within the context of the current study. Here, the objective is to demonstrate to the reader that similar approaches are indeed applicable to inorganic phosphate glasses. To our knowledge, this will be the first reported use of these equations with inorganic glasses as already mentioned in the main article.

Generally, two polymers (P1 and P2) are considered as fully miscible if only one glass transition is measured for their blend. Obviously this criterion is valid only if the T_g of each polymer is sufficiently different. Moreover they are miscible in all proportions if we obtained only one T_g for all the blend compositions. Furthermore, the variation of T_g with the composition of the polymer blends has been described by different theoretical equations. These equations are often derived from those established for random copolymers and the variation of T_g with concentrations of the monomers.

Among these equations, the most popular (and also the simplest) is the Fox equation[1]:

$$\frac{1}{T_g} = \frac{w_1}{T_{g1}} + \frac{w_2}{T_{g2}} \qquad (1)$$

where T_{gi} is the T_g of the polymer i and w_i is the weight fraction of polymer i. (w_2=1-w_1)

This equation does not contain any adjustable parameter. However it can fail in describing the T_g variation, especially for blends with strong interactions between both polymers. It always gives a negative deviation from the simple rule of mixtures.

The Gordon Taylor[2] (equation (2)) is another very popular equation which contains one adjustable parameter (K) to account for the interactions but it still fails in cases of very high interactions for which the deviation becomes positive or mixed (for instance positive for low P1 content and negative for high P1 content)

$$T_g = \frac{w_1 T_{g1} + K w_2 T_{g2}}{w_1 + K w_2} \tag{2}$$

From the consideration of the "free volume" hypothesis, Kelley and Bueche[3] have provided a rationalization of the Gordon-Taylor equation, which becomes:

$$T_g = \frac{\phi_1 T_{g1} + K_\alpha \phi_2 T_{g2}}{\phi_1 + K_\alpha \phi_2} \tag{3}$$

In equation (3) equation the volume fractions are used (but they can obviously be replaced by the weight fractions for materials of similar densities). Moreover the coefficient K_α is the ratio between the dilatation coefficients of free volume (α_f) of each polymer:

$$K_\alpha = \frac{\alpha_{f2}}{\alpha_{f1}} \tag{4}$$

These coefficients (α_f) represent the dilatation of the free volume fraction (f) for $T>T_g$ (f being fixed at f_g for $T<T_g$:

$$f = f_g + \alpha_f \left(T - T_g\right) \text{for } T>T_g \tag{5}$$

Kelley and Bueche[3] derived their relation by considering that:

1) The glass free volume fraction is the same for all polymers.

2) Each material supplies all but only its own free volume to the blend (i.e., the interactions P1-P1 and P2-P2 are equivalent to P1-P2). Thus there is no volume contraction or excess when blending.

Hence:

$$f = \phi_1 f_g + \alpha_{f1}\left(T - T_{g1}\right) + \phi_2 f_g + \alpha_{f2}\left(T - T_{g2}\right) \tag{6}$$

which can be rearranged into the following equation

$$\frac{f}{\alpha_{f1}} = \frac{f_g}{\alpha_{f1}} + \phi_1\left(T - T_{g1}\right) + \phi_2 K_\alpha \left(T - T_{g2}\right) \tag{7}$$

because $\phi_1 + \phi_2 = 1$

Moreover at $T= T_g$, $f= f_g$ so:

$$0 = \phi_1\left(T_g - T_{g1}\right) + \phi_2 K_\alpha \left(T_g - T_{g2}\right) \tag{8}$$

From equation (8), equation (3) is easily obtained.

Thus, the Kelley-Bueche equation[3] contains some physical explanation. However, it still does not account for specific interaction P1-P2. Therefore, another term was introduced considering a volume

contraction in the blend and the free volumes are not strictly additive anymore. Thus, equation (6) becomes:

$$f = \phi_1 f_g + \alpha_{f1}\left(T - T_{g1}\right) + \phi_2 f_g + \alpha_{f2}\left(T - T_{g2}\right) + K'\phi_1\phi_2 \quad (9)$$

where *K'* is used to take into account the volume contraction in the blend. Generally, it is a contraction (*K'*<0, order of magnitude 10^{-2}), because the good P1-P2 interactions are favorable for the miscibility). Thus equation (3) now becomes:

$$T_g = \frac{\phi_1 T_{g1} + K_\alpha \phi_2 T_{g2} + \dfrac{K'}{\alpha_{f1}}\phi_1\phi_2}{\phi_1 + K_\alpha \phi_2} \quad (10)$$

This last equation is very close to another very commonly used equation of Kwei[4] which contains two adjustable parameters and which is expressed by the weight fractions as follows:

$$T_g = \frac{w_1 T_{g1} + K w_2 T_{g2}}{w_1 + K w_2} + q w_1 w_2 \quad (11)$$

where *q* is correlated with the strength of the interaction in the binary blend.

Other equations have been derived from thermodynamic arguments. For example, Lu and Weiss[5] considered the variation of enthalpy due to the mixing. This enthalpy of mixing was expressed as a function of the Flory-Huggins[6] interaction parameter χ_{12}:

$$\Delta H_m\left(T\right) = \chi_{12} R T \phi_1 \phi_2 \quad (12)$$

The general equation takes the form (P2 is the polymer with the highest T_g):

$$T_g = \frac{w_1 T_{g1} + k w_2 T_{g2}}{w_1 + k w_2} + \frac{A w_1 w_2}{\left(w_1 + k w_2\right)\left(w_1 + b w_2\right)\left(w_1 + c w_2\right)^2} \tag{13}$$

with:

$$A = \frac{\chi_{12} R \left(T_{g1} - T_{g2}\right) c}{M_1 \Delta C p_1} \tag{14}$$

ΔCp_1 is the variation of the specific heat at the glass transition for the polymer 1.

$$c = \frac{\rho_1}{\rho_2}$$

$$b = \frac{M_1}{M_2}$$

ρ_i is the density of the polymer *i* and M_i is its molar mass per chain segment.

It must be pointed out that for blends with specific interactions P1-P2, the interaction parameter χ_{12} becomes negative and the coefficient *A* becomes positive (T_{g1} being lower than T_{g2})

$$k = \frac{\Delta C p_2 - w_1 \delta C p^l}{\Delta C p_1 - w_2 \delta C p^g} \tag{15}$$

where δCp is the specific heat change due to mixing (in glass or liquid state).

Since the interaction parameter χ_{12} can be composition dependent, this can lead to a complex form of the T_g variation with composition. Nevertheless, this enables equation (13) to predict both negative and positive curvatures.

Some simpler forms of equation (13) can be given for the following 3 types of interactions:

1) For only weak or no interactions between the polymers ($\chi_{12} \geq 0$), Lu and Weiss[5] obtained an equation predicting a negative deviation from the mixing rule, similar to that of Gordon Taylor already described:

$$T_g = \frac{w_1 T_{g1} + K_{lw} w_2 T_{g2}}{w_1 + K_{lw} w_2} \quad (16)$$

where $K_{lw} = \dfrac{\Delta Cp_2}{\Delta Cp_1} < 1$

Moreover, recalling the Simha-Boyer empirical rule[7] ($\Delta Cp_i T_{gi} = Cst$), the Fox equation (equation (1)) is obtained as depicted in the following equations:

$$T_g = \frac{w_1 T_{g1} + \dfrac{\Delta Cp_2}{\Delta Cp_1} w_2 T_{g2}}{w_1 + \dfrac{\Delta Cp_2}{\Delta Cp_1} w_2} = \frac{\Delta Cp_1 w_1 T_{g1} + \Delta Cp_2 w_2 T_{g2}}{\Delta Cp_1 w_1 + \Delta Cp_2 w_2}$$

Thus: $\dfrac{1}{T_g} = \dfrac{\Delta Cp_1 w_1 + \Delta Cp_2 w_2}{\Delta Cp_1 w_1 T_{g1} + \Delta Cp_2 w_2 T_{g2}} = \dfrac{1}{\Delta Cp T_g}\left(\Delta Cp_1 w_1 + \Delta Cp_2 w_2\right) = \dfrac{\Delta Cp_1 w_1}{\Delta Cp_1 T_{g1}} + \dfrac{\Delta Cp_2 w_2}{\Delta Cp_2 T_{g2}}$

2) For relatively weak interactions between the polymers, *i. e.* an effect of *k* exceeding that of *A*, Lu and Weiss[5] obtained the following equation which is still like the Gordon Taylor equation[2] in its form but with a coefficient K'_{lw} which is not an adjustable parameter anymore (it both contains *k* and *A* as the following equation (17) and equation (18) show):

$$T_g = \frac{w_1 T_{g1} + K'_{lw} w_2 T_{g2}}{w_1 + K'_{lw} w_2} \quad (17)$$

$$K'_{lw} = k + A/(T_{g2} - T_{g1}) \quad (18)$$

3) For relatively strong interactions between the polymers, equation (13) is used as is but δCp in equation (15) can be neglected. Additionally, the second term of equation (13) may be approximated by a Taylor series expansion to yield the Kwei equation (equation (11)).

Hence from the Lu and Weiss theoretical equation, the following empirical equations can be obtained: Fox for weak interactions[1]; Gordon-Taylor for moderate interactions[2]; and Kwei for stronger interactions[4].

Another approach was proposed by Couchman[8,9] considering the entropy variation in the blend instead of the enthalpy, leading to the following expression with the logarithm of the glass temperatures:

$$\ln T_g = \frac{\phi_1 \ln T_{g1} + K_c \phi_2 \ln T_{g2}}{\phi_1 + K_c \phi_2} \qquad (19)$$

where

$$K_c = \frac{\Delta Cp_2}{\Delta Cp_1}$$

Here, the Fox equation can be deduced by considering some approximations (i.e., Taylor series expansion of the logarithm and Simha-Boyer rule[7].

Another approach reported by Brekner *et al.*[10] can be mentioned. It is based on the numeration of the contacts P1-P1, P2-P2 and P1-P2 and the specific stored energy for each type of contact, resulting in the following equation:

$$T_g = \frac{w_1 T_{g1} + K_{bsc}\left(\frac{T_{g1}}{T_{g2}}\right) w_2 T_{g2}}{w_1 + K'_{bsc}\left(\frac{T_{g1}}{T_{g2}}\right) w_2} \qquad (20)$$

Note that the Fox equation and the Gordon-Taylor equation can be derived from equation (20). In the current work, the miscibility of the selected phosphate glass compositions of significantly different glass transition temperatures and compositions are studied and the obtained results interpreted and analyzed according to the preceding theoretical considerations.

References

[1] Fox, T. G. Influence of diluent and of copolymer composition on the glass temperature of a polymer system. *Bull. Am. Phys. Soc.* **1**, 123-128 (1956).

[2] Gordon, M. & Taylor, J. S. Ideal copolymers and the second-order transitions of synthetic rubbers. I. Noncrystalline copolymers. *J. Appl. Chem.,* 1952, **2**, 493-500.

[3] Kelley, F. N. & Bueche F. Viscosity and glass-temperature relations for polymer-diluent systems. *J. Polym. Sci*. **50,** 549–556 (1961).

[4] Kwei, T. K. The effect of hydrogen bonding on the glass transition temperatures of polymer mixtures. J. *Polym. Sci., Polym. Lett. Ed.* **22**, 307-313 (1984).

[5] Lu, X. & Weiss, R. A. Relationship between the glass transition temperature and the interaction parameter of miscible binary polymer blends. *Macromolecules*, **25**, 3242-3246 (1992) (Corrections in *Macromolecules* 1993, **26**, 248).

[6] Flory, P. J. *Principles of Polymer Chemistry* (Cornell Univ. Press, Ithaca, NY, 1953).

[7] Boyer, R. F. & Simha, R. Relation between expansion coefficients and glass temperature. Reply to comments. *J. Polym. Sci. Polym Lett*. **11**, 33-44 (1973).

[8] Couchman, P. R. Compositional variation of glass transition temperatures. 2. Application of the thermodynamic theory to compatible polymer blends. *Macromolecules* **11**, 1156-1161 (1978).

[9] Couchman, P. R. The composition-dependent glass transition: relations between temperature, pressure, and composition. *Polym. Eng. Sci.* **24**, 136-143 (1984).

[10] Brekner, M-J., Schneider, H. A. & Cabtow, H-J. Approach to the composition dependence of the glass transition temperature of compatible polymer blends. 1. *Polymer*, **29**, 78-85 (1988).
